# Online Medication Abortion Direct-to-Patient Fulfillment Before and After the *Dobbs v Jackson* Decision

**DOI:** 10.1001/jamanetworkopen.2024.34675

**Published:** 2024-10-04

**Authors:** Caila Brander, Jessica Nouhavandi, Terri-Ann Thompson

**Affiliations:** 1 Ibis Reproductive Health, Cambridge, Massachusetts; 2 Honeybee Health, Culver City, California

## Abstract

**Importance:**

Online pharmacies have emerged as stakeholders in abortion care since the US Food and Drug Administration (FDA) relaxed in-person dispensing requirements in 2020. The role of online pharmacies in dispensing abortion medications following the *Dobbs v Jackson Women’s Health Organization* decision on June 24, 2022, is understudied.

**Objective:**

To describe medication abortion prescription fulfillment patterns for 1 online pharmacy 1 year before and after the *Dobbs v Jackson* decision, considering patient, prescriber, and state policy characteristics.

**Design, Setting, and Participants:**

This cross-sectional study assesses deidentified medication abortion prescription fulfilment data from 1 online pharmacy. Prescribers sent prescription requests to the online pharmacy, which dispensed abortion medications to patients by mail. The study sample included prescription requests fulfilled by the online pharmacy between June 20, 2021, and June 24, 2023, for patients aged at least 18 years who received the combined medication abortion regimen. Data were analyzed from July 2023 to July 2024.

**Exposures:**

The US Supreme Court *Dobbs v Jackson* decision on June 24, 2022.

**Main Outcomes and Measures:**

Patient and prescriber characteristics are described, including patient age, state to which the prescription was sent, medications prescribed, and prescribing prescriber’s clinic care modality (in-person only, hybrid [in-person and virtual], and virtual only). States were grouped according to the Guttmacher Institute classification of policy support for abortion (most or very supportive, somewhat supportive, and somewhat restrictive). Fulfillment trends were disaggregated by prescriber modality and state policy environment; 2 states with similar telehealth but differing coverage policies (Illinois and Colorado) were compared.

**Results:**

The dataset included 87 942 observations. Most prescriptions were sent to individuals younger than 30 years (57.1%), with a mean (SD) age of 28.7 (6.4) years. Throughout the study period, the greatest volume of prescription requests came from states with most or very supportive policies and from prescribers at virtual-only platforms. More prescriptions were sent in the year after *Dobbs v Jackson* (daily mean [SD], 88.5 [47.2] prescriptions in March 2022 vs 201.5 [97.5] prescriptions in March 2023) with fulfillment spikes following the *Dobbs v Jackson* leak on May 2, 2022, and decision on June 24, 2022. State policy contexts mirrored the overall trends, while prescriber modality trends were unique, with a big spike in fulfillment at 12 weeks after the *Dobbs v Jackson* decision for hybrid clinics compared with in-person-only clinics and telehealth-only platforms, which saw their largest spikes in mean daily prescription fulfillment the week immediately after *Dobbs v Jackson*. Illinois and Colorado had similar fulfillment trends, with spikes immediately following the *Dobbs v Jackson* decision and overall higher fulfillment after *Dobbs v Jackson*, with a daily mean (SD) of 10.5 (7.0) prescriptions in Illinois and 8.8 (5.7) prescriptions in Colorado in March 2022 versus 26.6 (13.6) prescriptions in Illinois and 16.7 (10.1) prescriptions in Colorado in March 2023.

**Conclusions and Relevance:**

These findings illustrate the increasingly critical role online pharmacies play in direct-to-patient abortion care provision in the US and the strong linkages between virtual-only prescribers and online pharmacies. These findings suggest that barriers to accessing online pharmacies for abortion care should be removed.

## Key Points

**Question:**

How did prescription fulfilment for direct-to-patient medication abortion provision change for 1 online pharmacy after the *Dobbs v Jackson Women’s Health Organization* decision, considering patient, prescriber, and state policy characteristics?

**Findings:**

In this cross-sectional study including 87 942 fulfilled prescriptions, there was an overall increase in prescription fulfilment for direct-to-patient medication abortions in the year after the *Dobbs v Jackson* decision compared with the year prior, with spikes in prescription fulfillment following the *Dobbs v Jackson* leak and the *Dobbs v Jackson* decision itself. Most medication was prescribed by virtual-only platforms and sent into states with supportive abortion policies.

**Meaning:**

These findings suggest that online pharmacies play an increasingly critical role in direct-to-patient medication abortion provision, especially with virtual-only platforms.

## Teaser

This cross-sectional study describes trends in fulfillment patterns for medication abortion prescriptions from 1 online pharmacy 1 year before to 1 year after the US Supreme Court *Dobbs v Jackson Women’s Health Organization* decision.

## Introduction

In 2020, the US Food and Drug Administration (FDA) removed in-person and clinician-only dispensing requirements as part of the Risk Evaluation and Mitigation Strategy (REMS) attached to mifepristone, the first drug in the combined medication abortion (MA) regimen.^1^ Prior to this decision, the only direct-to-patient telehealth abortion services, ie services that allow clients to have a remote consultation with a health care practitioner before having MA delivered to their home, were through groups like Women on Web^2^ and Aid Access.^3^ While demonstrably safe, effective, and acceptable to patients,^4,5^ these services existed outside the purview of the formal US health care system, which created legal and practical drawbacks.^6,7^ The FDA’s decision allowed direct-to-patient provision of MA through the formal health care system for the first time.

Direct-to-patient telehealth provision of MA involves the prescriber sending an MA prescription to an online pharmacy, which then sends the medications to the patient’s preferred address. Some patients may use virtual mailboxes, post office boxes, and forwarding addresses that allow pills to be rerouted to their home address.^8^ Online pharmacies offer a simple “one-stop-shop” solution for telehealth prescribers, who can count on the pharmacy to dispense and ship MA wherever abortion is provided. Brick-and-mortar clinics can also use online pharmacies to fulfill MA prescriptions, eliminating the burden of stocking, storing, and dispensing MA.

In the years that followed the FDA decision, surveillance efforts, such as the Society of Family Planning’s #WeCount initiative^9^ as well as the Guttmacher Institute’s abortion provider census^10^ and monthly abortion provision study,^11^ reported an increase in abortion provision, a shift from procedural to medication abortion as the most common abortion procedure in the US, and an increase in direct-to-patient telehealth abortions. In 2023, approximately 63% of abortions were medication abortions, up from 53% in 2020,^12^ and provision by virtual-only platforms increased by 137% in the year after the *Dobbs v Jackson Women’s Health Organization* decision.^9,13^ Furthermore, a study by Koenig et al^14^ found that virtual clinic abortion practitioners proliferated after *Dobbs v Jackson*, moving from 11 virtual clinics providing care in 26 states and Washington, District of Columbia, in September 2022 to 20 virtual clinics providing care in 27 states and Washington, District of Columbia by June 2023.^14^

The dramatic increase in direct-to-patient telehealth medication abortion provision has direct implications for online pharmacies that provide MA. However, to our knowledge, no studies have described the role of online pharmacies in direct-to-patient abortion provision or how that role may have shifted after the *Dobbs v Jackson* decision. In this study, we describe fulfillment patterns for 1 online pharmacy over a 2-year period with special emphasis on the *Dobbs v Jackson* decision and consider prescriber care modalities and state policy contexts.

## Methods

This cross-sectional study was approved by the Allendale Investigational Review Board, which waived the requirement for informed consent because only deidentified data were used. This study is reported following the Strengthening the Reporting of Observational Studies in Epidemiology (STROBE) reporting guideline for cross-sectional studies.

We assessed prescription fulfillment records from patients aged 18 to 49 years who received the combined MA regimen of mifepristone and misoprostol from 1 online pharmacy. The online pharmacy received requests for MA prescriptions and (sometimes) medications to manage abortion symptoms following a patient’s visit. The pharmacy sent prescriptions to the patient’s preferred location. We received deidentified data from the pharmacy for prescription requests fulfilled between June 20, 2021, and June 24, 2023. The data included a limited number of predetermined variables: patient age, state the prescription was sent to, date the prescription was received and fulfilled, medications sent, and prescriber’s clinic care modality.

Patient age was regrouped into 6 categories (18-24, 25-29, 30-34, 35-39, and 40-49 years). States were regrouped by Census region^15^ and abortion policy environment using classifications from the Guttmacher Institute .^16^ The online pharmacy only provided care to states where direct-to-patient abortion services were legally permitted. Therefore, there are no observations for states with abortion bans (most restrictive) in the dataset. Additionally, we excluded observations for states with fewer than 500 observations, which included 4 states classified as restrictive or very restrictive (total 641 observations). Thus, we include observations from states categorized as somewhat restrictive, somewhat protective, and very or most protective (combined). We denote the prescriber’s clinic care modality by the online pharmacy’s classification: in-person–only clinic, hybrid clinic (provides both in-person and virtual services), or virtual-only platform. To assess whether prescribers prescribed supplementary medications offered by the online pharmacy, we created binary variables (prescribed or not prescribed) based on whether a prescription contained pain relief (ibuprofen, naproxen, acetaminophen) or antinausea (ondansetron, promethazine, prochlorperazine, metoclopramide) medications.

Prescription fulfillment records provided by the pharmacy included the date that the prescriptions were sent; we recategorized this into calendar weeks (Sunday to Saturday) and months. We calculated the mean daily prescriptions fulfilled by the online pharmacy for each week and month. We then graphed the daily mean by week for the duration of the study and described visible trends in the graphs. Given the potential implications of the *Dobbs v Jackson* decision leak on May 2, 2022,^17^ and the announcement of the final decision on June 24, 2022,^13^ on abortion provision,^18^ we also assessed changes based on these dates. We graphed fulfillment trends stratified by state policy context and prescriber modality. Finally, we included a case study comparing Illinois and Colorado, 2 states that have similar telehealth policies, such as support for asynchronous care models, but differ on Medicaid coverage of abortion^19^ to see what other factors might be associated with increases in direct-to-patient provision and thus online pharmacy use.

### Statistical Analysis

We used Stata version 15 (StataCorp) to calculate descriptive statistics (percentages, means) and time series graphs. Data were analyzed from July 2023 to July 2024

## Results

Our sample included a total of 87 942 observations (Table). Most prescriptions were for individuals younger than 30 years (57.1%; mean [SD] age, 28.7 [6.4] years), were sent to postal addresses in the West (51.9%), and included medication to address pain (49.8%) or nausea (61.5%) adverse effects.

**Table.** Patient Demographics in a Sample of Direct-to-Patient Prescriptions Sent From 1 Online Pharmacy, 1 Year Before Until 1 Year After the *Dobbs v Jackson* Decision

Characteristic	No. (%) (N = 87 942)
Patient age, y	
18-19	5433 (6.2)
20-24	21 406 (24.3)
25-29	23 410 (26.6)
30-34	20 071 (22.8)
35-39	12 450 (14.2)
40-49	5202 (5.9)
Mean (SD)	28.7 (6.4)
Census region the prescription was sent to	
Midwest	14 142 (16.1)
Northeast	19 442 (22.1)
South	8694 (9.9)
West	45 694 (51.9)
State policy environment^a^	
Most or very protective	45 941 (52.2)
Somewhat protective	30 229 (34.4)
Somewhat restrictive	11 802 (13.4)
Clinic care modality	
Hybrid (virtual + in-person)	12 853 (14.6)
In-person only	6554 (7.5)
Virtual only	68 565 (77.9)
Additional prescriptions	
Nausea relief	54 126 (61.5)
Pain relief	43 825 (49.8)

^a^ Most and very protective states include Oregon, California, Minnesota, New Jersey, New Mexico, New York, and Vermont. Protective states include Alaska, Colorado, Connecticut, District of Columbia, Hawaii, Illinois, Massachusetts, Maryland, Maine, and Washington. Some restriction states include Delaware, Michigan, New Hampshire, Nevada, Rhode Island, Virginia, and Wyoming.

**Figure 1.**
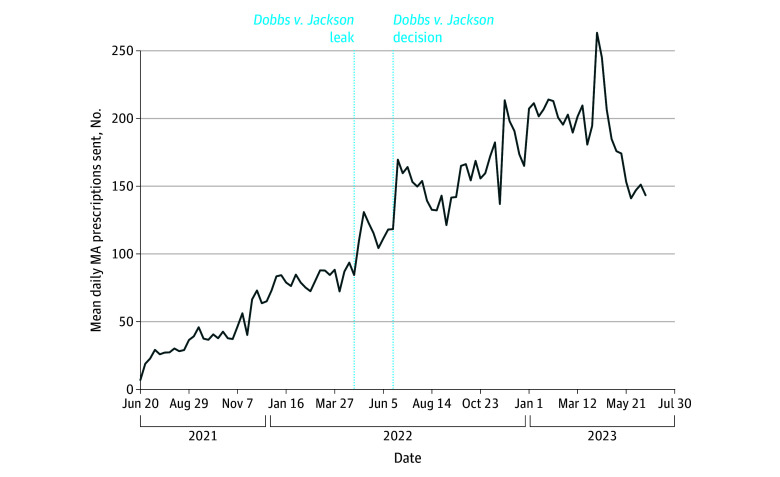
Mean Daily Prescription Requests Fulfilled by the Online Pharmacy Each Week Between June 20, 2021, and June 24, 2023 (N = 87 942) MA indicates medication abortion.

Figure 1 presents the mean daily prescriptions per week from 1 year before until 1 year after the *Dobbs v Jackson* decision. There was a noticeable spike in fulfillment in the week immediately following both the leak of the *Dobbs v Jackson* decision and the decision itself. The week before the leak, the online pharmacy sent a mean (SD) of 84.4 (57.0) MA prescriptions per day. In the week after the *Dobbs v Jackson* leak, this increased to 130.9 (75.4) per day. Following the *Dobbs v Jackson* decision, fulfillment rose to a mean (SD) of 169.6 (84.8) prescriptions per day. The initial spikes in fulfillment decreased slightly in the subsequent weeks. However, approximately 1 month after the *Dobbs v Jackson* decision, medication fulfillment began to increase again, eventually surpassing and sustaining a fulfillment level higher than the initial spikes after the leak and after the decision. The online pharmacy fulfilled more prescriptions in each month after *Dobbs v Jackson* compared with the same month the year prior. For example, in March 2022, the online pharmacy dispensed a mean (SD) of 88.5 (47.2) MA prescriptions per day; by March 2023, this number had increased to 201.5 (97.5) MA prescriptions per day. Another spike in fulfillment appeared 40 weeks after the *Dobbs v Jackson* decision (April 10-16, 2023).

**Figure 2.**
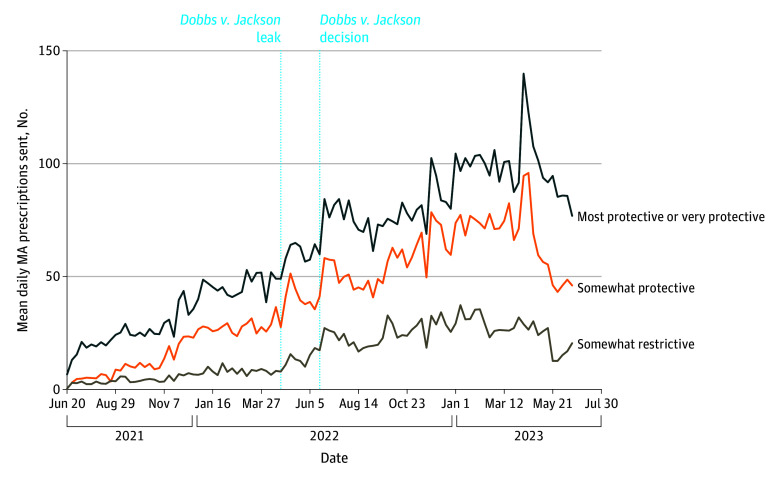
Mean Daily Prescription Requests Fulfilled by the Online Pharmacy Each Week Between June 20, 2021, and June 24, 2023, by State Abortion Policy Environment (N = 87 942) MA indicates medication abortion.

We disaggregated prescription fulfillment trends by prescriber care modality in Figure 2. The online pharmacy was used most by virtual-only platforms during the entire study period. Trends in overall fulfillment were mirrored by the virtual-only platforms. However, 12 weeks after the *Dobbs v Jackson* decision, there was an increase in prescription fulfillments from hybrid clinics (mean [SD], 10.3 [7.9] vs 28.6 [20.4] daily prescriptions), followed by a steep decline before the end of the study period. The number of prescriptions fulfilled by in-person-only clinics increased steadily after *Dobbs v Jackson*, with a mean (SD) of 3.9 (2.5) daily prescriptions in March 2022 vs 21.6 (10.7) by March 2023.

**Figure 3.**
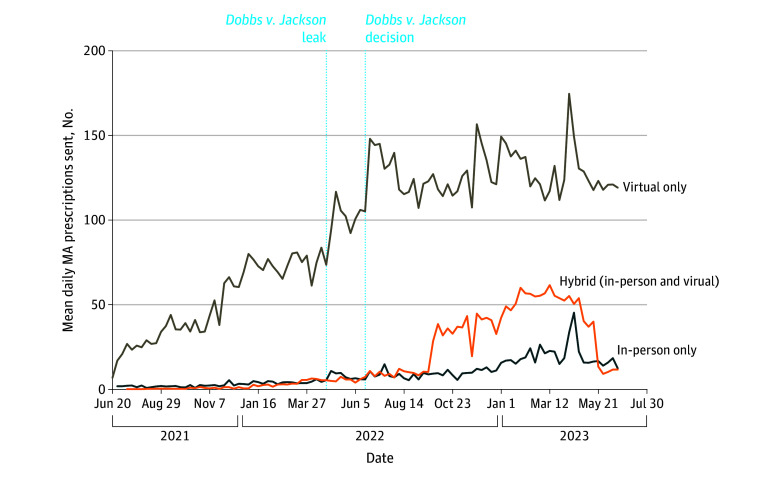
Mean Daily Prescription Requests Fulfilled by the Online Pharmacy Each Week Between June 20, 2021, and June 24, 2023, by Prescriber Practice Modality (N = 87 942) MA indicates medication abortion.

In Figure 3, fulfillment trends are disaggregated by state abortion policy environments. The online pharmacy sent most prescriptions to most or very protective states, then somewhat protective states, and finally the least to somewhat restrictive states both before and after the *Dobbs v Jackson* decision. The general trends for all 3 policy environments were similar. However, the surge observed in 40 weeks after *Dobbs v Jackson* in Figure 1 only appears for most or very protective or somewhat protective states and not in somewhat restrictive states.

**Figure 4.**
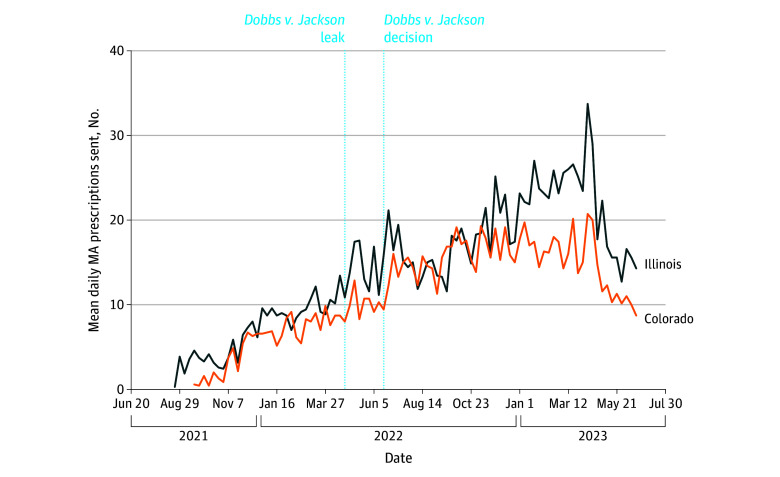
Mean Daily Prescription Requests Fulfilled by the Online Pharmacy Each Week Between June 20, 2021, and June 24, 2023 in Colorado and Illinois MA indicates medication abortion.

Our 2-state comparison of trends is presented in Figure 4. Trends in fulfillment were similar between Colorado and Illinois, with initial spikes immediately following the *Dobbs v Jackson* decision and a post–*Dobbs v Jackson* decision baseline in both states that far surpassed pre–*Dobbs v Jackson* fulfillment. Fulfillment decreased more steadily in Illinois after the initial post-*Dobbs v Jackson* spike. As time progressed, fulfillment to Colorado declined slightly while fulfilment in Illinois continued to increase. Despite these differences, fulfillment fluctuations immediately before and after the *Dobbs v Jackson* decision were largely similar between the 2 states and overall prescription fulfillment was higher after *Dobbs v Jackson*, with a daily mean (SD) of 10.5 (7.0) prescriptions in Illinois and 8.8 (5.7) prescriptions in Colorado in March 2022 versus 26.6 (13.6) prescriptions in Illinois and 16.7 (10.1) prescriptions in Colorado in March 2023.

## Discussion

This cross-sectional study using data from 1 online pharmacy found an increase in MA prescription fulfilment requests between June 2021 and June 2023. The online pharmacy dispensed more MA in the first year following the *Dobbs v Jackson* decision compared with the year before, showing this relatively new stakeholder to the abortion provision space played an increasingly important role after June 24, 2022. Across the entire study period, many orders included medications to manage abortion symptoms, and most orders were prescribed by prescribers at virtual-only platforms. When disaggregated by state abortion policy environments, all 3 contexts showed very similar patterns. However, fulfillment patterns disaggregated by prescriber modality were unique for all 3 modalities. Finally, a comparison of 2 states with similar telehealth and abortion environments, except Medicaid coverage of abortion, revealed similar fulfilment trends.

These findings align with pre–*Dobbs v Jackson* trends, such as greater utilization of MA,^20^ and post–*Dobbs v Jackson* trends, such as an increase in virtual-only platforms for abortion care.^9^ Additionally, patients’ ages aligned with abortion patient demographics nationally.^21^ Comparing the trends observed in this study to #WeCount, which counted the total number of abortions provided in the US just before and more than a year after *Dobbs v Jackson*, we observe some differences. The spikes in abortion provision immediately following the *Dobbs v Jackson* leak or decision were smaller within the #WeCount report than those observed in our study.^22^ This may be due to the fact that #WeCount reported abortions by month rather than by week, as done in this study, thereby potentially blunting the effects of such a spike. Furthermore, our study contains a greater proportion of virtual-only prescribers, and the spike that followed the leak and the *Dobbs v Jackson* decision was reflected most prominently by this prescriber type. This suggests that virtual-only platforms and online pharmacies that dispense medication to their patients may have played an even greater role than previously understood in accommodating shifts in the abortion provision landscape in the wake of the *Dobbs v Jackson* leak and decision. A similar pattern of spikes and declines in requests to 1 telemedicine service was also observed after Texas passed the SB-8 abortion ban in September 2021. A study by Aiken et al^23^ theorized that the initial spike may have been due to clinic cancellations or confusion about eligibility for abortion and that the decline that followed was due to the sustained efforts of advocacy groups and abortion funds connecting abortion seekers to out-of-state care. It is quite possible something similar happened nationally after the *Dobbs v Jackson* decision. Similar to #WeCount, we also observed an increase in prescription requests at 40 weeks after the *Dobbs v Jackson* decision (April 10-16, 2023). Since our study is not representative of overall abortion provision, #WeCount data may be more useful in determining what may have caused that spike.

The unique trends that appear in prescriber modality may be due, in part, to the nature of our study design. During the 2 years we assessed, the number of prescribers that the online pharmacy worked with was not constant. Changes in overall prescription volume could indicate either the same set of partners prescribing increasing amounts of medication or an increase or decrease in the number of prescribers using the online pharmacy to fulfill their prescription requests. Delayed increases in prescription requests from hybrid clinics and drastic declines in fulfilment requests could be explained by some prescribers needing more time to add telehealth to their models or to 1 or more prescribers with larger patient volumes no longer using the online pharmacy, respectively.

Several factors could explain the similarities and differences observed in our state comparison for prescription fulfilments. The higher spikes in Illinois following the Dobbs leak and decision and higher fulfilment requests overall may be due to differences in abortion rates and Medicaid coverage between the states. Illinois has a higher abortion rate than Colorado^9,11^ and has seen an increase in abortion seekers from out-of-state, especially after bans went into effect in neighboring states.^11^ Additionally, research has shown that Medicaid coverage of abortion increases abortion access.^24,25^ However, the overall similarity between the states could suggest that policies that facilitate telehealth provision, bolstered by the relatively lower cost of telehealth services vs in-clinic care, may be bigger drivers of direct-to-patient telehealth utilization. Further research on the broader abortion ecosystem within the US is needed to better understand the influence of Medicaid coverage and other policies on direct-to-patient telehealth medication abortion utilization and access.

Mifepristone still carries REMS requirements issued by the FDA for medications with serious safety concerns.^26^ However, MA is safe and effective whether or not it is administered by a physician.^5^ There is increasing evidence to support adapting the REMS to allow pharmacies to directly counsel and dispense MA.^27,28^ These could be critical tools in the expansion of abortion provision beyond abortion clinics, and one in which online pharmacies are poised to play a leading role.

The role of online pharmacies provides intriguing lines of inquiry for better understanding and meeting patient preferences for support during and after a medication abortion. More than half of MA prescription requests in this study included additional prescriptions. Patients who received prescription-strength antipain or antinausea medication may have experienced less discomfort than if relying on over-the-counter medications, and direct-to-patient provision streamlines this option. Although not explored in this study, online pharmacies also have the capacity to dispense birth control medication to patients seeking abortion care, including emergency contraception. Future studies could assess whether unique benefits of direct-to-patient provision for supplementary prescriptions or ongoing reproductive health care support impact the quality of abortion experiences compared with other clinical care modalities.

### Limitations

This study has a few limitations. It is not representative of all abortions or direct-to-patient prescriptions, which means that the trends observed here are not indicative of overall post–*Dobbs v Jackson* trends. Given the inclusion criteria for the study, the dataset does not reflect total prescription volume for the online pharmacy during the study period. Demographic information on the patients receiving the prescriptions was limited. While we know the states where the medications were shipped, this may not reflect the states where patients lived and thus cannot be interpreted as definitive for the reach of online pharmacies. Additionally, the number of prescribers fluctuated during the time period. As a result, it is not possible to distinguish whether fluctuations in prescription volume were necessarily due to prescribing changes among the same group of prescribers or due to shifts in partnership with the online pharmacy. Nevertheless, to our knowledge, this is the first study to assess MA prescription trends from any online pharmacy in the post–*Dobbs v Jackson* era. Our analysis demonstrates the growing role of this new stakeholder in the abortion landscape and offers several lines of inquiry for future research.

## Conclusions

This cross-sectional study of prescription fulfillment before and after the US Supreme Court *Dobbs v Jackson* decision for 1 online pharmacy found an overall increase in prescription fulfillments over time, with spikes in fulfillment immediately following the *Dobbs v Jackson* leak and decision. Most prescriptions were sent to patients who received care from virtual-only platforms. The online pharmacy played a pivotal role in abortion care during momentous changes to the US policy landscape; barriers to direct-to-patient care, such as telehealth restrictions and the FDA’s remaining REMS restrictions, should be removed to create greater access to this mode of care.

## Notes

Supplement.
Data Sharing Statement
