# Supplementary material for: Online Medication Abortion Direct-to-Patient Fulfillment Before and After the Dobbs v Jackson Decision
Source: JAMA Netw Open. 2024 Oct 4;7(10):e2434675. doi: 10.1001/jamanetworkopen.2024.34675 (PMC11452820; doi:10.1001/jamanetworkopen.2024.34675)
Supplement: Supplement. — Data Sharing Statement [file jamanetwopen-e2434675-s001.pdf]

Data Sharing Statement

Brander. Online Medication Abortion Direct-to-Patient Fulfillment Before and After Dobbs v Jackson Decision. *JAMA Netw Open*. Published October 04, 2024. doi:10.1001/jamanetworkopen.2024.34675

Data
Data available: No

Additional Information
Explanation for why data not available: To preserve the anonymity of the providers and patients served by the online pharmacy, the dataset for this study will not be made publicly available.
